# Discrimination of Different Species of Dendrobium with an Electronic Nose Using Aggregated Conformal Predictor

**DOI:** 10.3390/s19040964

**Published:** 2019-02-25

**Authors:** You Wang, Zhan Wang, Junwei Diao, Xiyang Sun, Zhiyuan Luo, Guang Li

**Affiliations:** 1 State Key Laboratory of Industrial Control Technology, Institute of Cyber Systems and Control, Zhejiang University, Hangzhou 310027, China; king_wy@zju.edu.cn (Y.W.); 11732003@zju.edu.cn (Z.W.); djw@zju.edu.cn (J.D.); 21532085@zju.edu.cn (X.S.); 2 Computer Learning Research Centre, Royal Holloway, University of London, Egham Hill, Egham, Surrey TW20 0EX, UK; zhiyuan@cs.rhul.ac.uk

**Keywords:** dendrobium, conformal prediction, electronic nose, aggregated

## Abstract

A method using electronic nose to discriminate 10 different species of dendrobium, which is a kind of precious herb with medicinal application, was developed with high efficiency and low cost. A framework named aggregated conformal prediction was applied to make predictions with accuracy and reliability for E-nose detection. This method achieved a classification accuracy close to 80% with an average improvement of 6.2% when compared with the results obtained by using traditional inductive conformal prediction. It also provided reliability assessment to show more comprehensive information for each prediction. Meanwhile, two main indicators of conformal predictor, validity and efficiency, were also compared and discussed in this work. The result shows that the approach integrating electronic nose with aggregated conformal prediction to classify the species of dendrobium with reliability and validity is promising.

## 1. Introduction

With the ever-increasing popularity of alternative herbal medicines on international stage, traditional Chinese medicine, one of the key sources of global herbal medicines, is also gaining more acceptance and worldwide renown. Dendrobium, recorded as “the first of nine Chinese fairy herbs” in Chinese history [1], has been widely used as herbal medicine and functional food in Asia. Although not ubiquitous, this medicine and its associated values have been emphasized by many scholars. Over the past years, recent studies have shown that dendrobium enjoys such functions as anti-cancer activity [2,3,4,5,6,7], immunomodulating activity [7,8,9,10], anti-diabetic activity [7,11,12,13], hepatoprotective activity [7,14,15,16], anti-inflammatory activity [7,17,18,19] and so on, when used as herbal medicines. With paramount medicinal value, the prices of certain species of dendrobium have even reached US$3000/kg [7,20]. Since there are too many different species of dendrobium with varying medicinal values, it is of great significance to find reliable methods to make discrimination. Since dendrobium is always sold after dehydration processing or even milling process in medicinal material market, it is of great difficulty to classify the species. The traditional identification method is analysis by pharmacist, which is expensive and too dependent on personal experience. Several methods, such as mass spectrometry (MS) [21,22,23], high performance liquid chromatography (HPLC) [1,24] and infrared spectroscopy(IRS) [1,25], are reported as valid methods for dendrobium classification. These methods are reported to classify 3–6 species ranging at accuracy of 88–90% focusing on the different compositions of different dendrobium. However, the methods applied by scholars in these articles generally necessitate long identification time, complicated experiment and very high price instrument.

Electronic nose (E-nose), designed by simulating the mechanism of human olfaction, has been applied to odor analysis in many fields such as environment quality evaluation [26,27,28,29,30,31,32], medical diagnosis [33,34,35,36] and food evaluation [37,38,39,40,41,42,43]. It reflects the nature of samples through detecting the volatile organic compounds with the advantages of high efficiency and low cost. As the sensor array of E-nose is often complex for comprehensive detection, the method to analyze E-nose data is significant. Thus far, some algorithms have been applied for E-nose prediction successfully such as support vector machine [41], k-nearest neighbors [44,45], artificial neural network [31,46] and so on. In our early work, a framework named conformal prediction (CP) has been applied for E-nose analysis to provide confidence level and make prediction more reliable [45]. To improve the efficiency of CP, an optimized framework named inductive conformal prediction (ICP) was presented by dividing traditional training set into training set and calibration set with probable sacrifice of accuracy. Therefore, aggregated conformal prediction (ACP) is set up to improve the accuracy of ICP recently [47,48,49,50,51].

In this study, a self-assembled electronic nose combined with aggregated conformal prediction to classify 10 common species of dendrobium with reliability and validity was introduced. The details of the experiment and E-nose equipment are illustrated in Section 2. The data analysis methods are introduced in Section 3 and the results of the experiment are manifested in Section 4. Finally, we draw the conclusion of our research in Section 5.

## 2. Experimental

### 2.1. Sample Preparation

In this study, 10 common species of dendrobium were purchased as the experimental materials from Hangzhou Medicinal Material Market (Hangzhou, China) randomly. The details of these materials are presented in Table 1. Ten grams of the material from every species of dendrobium were considered one specimen, and 50 dendrobium specimens for each species were made totally.

To let the volatile gas get better saturated, all 500 dendrobium specimens were ground into powder and hermetically sealed into empty wide-mouth glass bottles separately. The bottles had been cleaned with standard air for 30 min before sample preparation. Then, we marked the bottles and placed them into an electric stove at the temperature of 50 ${}^{\circ }$C and let the volatile gas be saturated for 10 h, so that the volatile organic compounds in every sample could volatilize sufficiently. Finally we used the headspace volatile organic compounds as characteristic gas samples for electronic nose analysis. Every specimen was extracted the characteristic gas samples only once.

### 2.2. Electronic Nose Analysis

**Figure 1:**
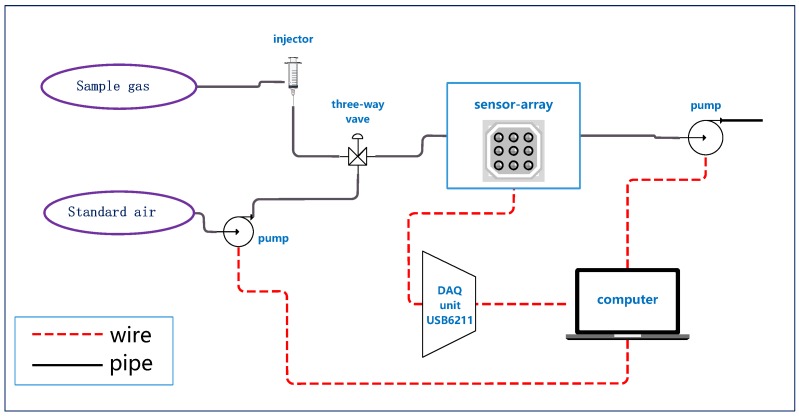
The structure of the self-assembled E-nose.

An electronic nose (E-nose) equipped with 16 metal-oxide semi-conductive sensors was used to analyze the dendrobium gas samples. All sensors were bought from Figaro Engineering Inc. (Osaka, Japan). The sensors selected do not have excessive specificity towards one type of gas, which could respond to the volatile gas compounds emanated by dendrobium specimens including many different types of alkalies, ethers, phenols and aldehydes. It is appropriate for herbal medicine odor analysis due to the complicated composition to find corresponding pattern of different species of dendrobium. Table 2 lists the specific affinity of each sensor. All sensors, fixed on a circuit board, were placed in a 200 mL box made by poly-tetra-fluoroethylene (PTFE), which served as a reaction chamber. Some of the sensors were fixed repetitively with different positions in the chamber in case of damaging. There were two fixed mini vacuum pumps with the vacuum of 80 kPa to provide power for standard air to clean the sensors and box with a flow at 1 L/min. Such flow can wash the box and sensors clean in 100–200 s without damaging the system. To control the switch between target gas and standard washing air, a three-way valve was equipped. A data acquisition (DAQ) unit USB6211 was used to record the response of all sensors, which was produced by National Instruments Inc. (Austin, TX, USA). We also provided a voltage of 5 V DC to heat the sensors and guarantee the best performance of the E-nose, which is recommended by Figaro Engineering Inc. Finally, a computer was used to provide electricity and control the E-nose. The overall structure of the E-nose is shown in Figure 1.

**Figure 2:**
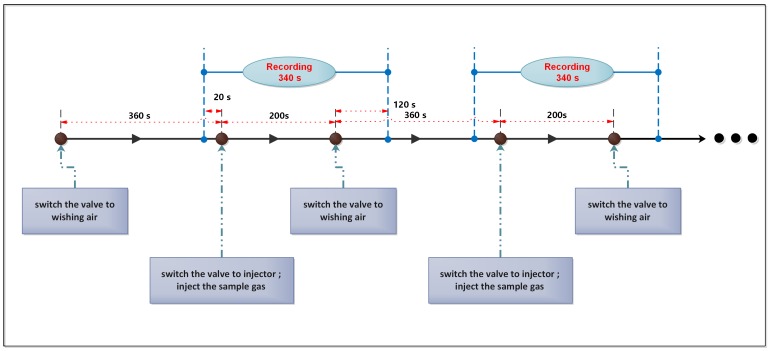
The procedure of measurement (including two of measurements).

The E-nose analysis was conducted at the temperature of 23 ± 2 ${}^{\circ }$C and humidity of 60±10%. The process of E-nose measurement is shown as follows. First, switch the valve to the standard air to wash all the sensors with an air flow of 1 L/min for 360 s to clean the sensors and let the sensor responses return to baseline. Second, extract 10 mL head-space gas from the top of the gas sample bottle that is placed in the electronic stove using an injector. Third, stop the standard air flow, switch the valve and inject the sample gas into the reaction chamber. The sample gas would diffuse in the chamber freely for 200 s and react until obtaining stable sensors reading. The time between the second step and the third step is less than 10 s. After that, switch the valve and change to the standard air flow, so that the sensors and the chamber could be washed clean to prepare for the next measurement. In each measurement, we recorded the data from all sensors for 340 s, including 20 s before the injection of sample gas, 200 s of reaction time and the first 120 s of cleaning time. The sampling frequency was 100 Hz. All 500 gas samples were injected to make analysis in a random order to reduce environmental disturbances and the experiment lasted for beyond one month to guarantee the reproducibility of measurement. The process of measurement is depicted in Figure 2, which describes two cycles of measurements.

## 3. Data Analysis Method

### 3.1. Data Preprocessing

**Figure 3:**
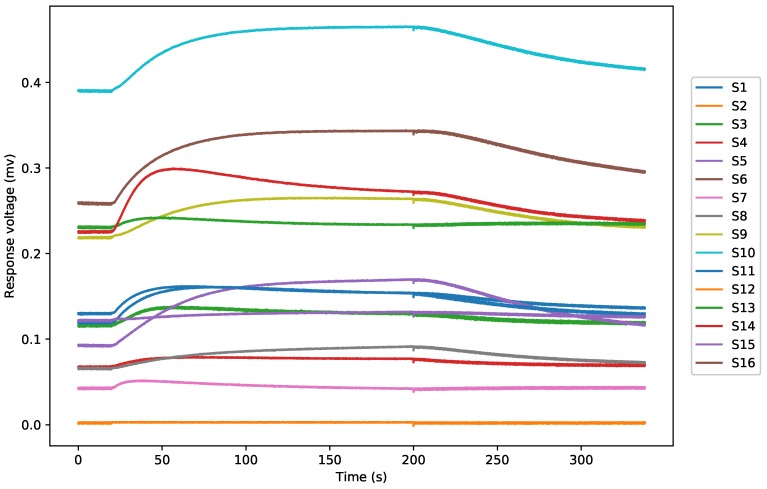
A typical E-nose response curves from a dendrobium gas sample.

In these studies, all analyses were performed using Python 3.5.2. A typical response curve of all sensors to a sample is shown in Figure 3, which shows the change of voltage from all channels (S2 obtained a similar signal as S3 and S6 obtained a similar signal as S7, thus both S2 and S6 are hidden in the figure being covered by S3 and S7).

Firstly, we changed the gas-sensor reaction signals to resistance signals. To reduce the effect stemming from sensor drift, the data were calibrated separately as follows:

(1) $$R=\left(R_{sample}-R_{baseline}\right)/R_{baseline}$$

where $R_{sample}$ is the original resistance data and $R_{baseline}$ is the baseline value gained by calculating the average value from the first 20 s of data.

### 3.2. Feature Extraction

Feature extraction is of great importance in classification problem when using E-nose since the group of features represents the whole response of all sensors. The major target of feature extraction is to get robust and concentrated information from sensor responses with less redundancy [52].

To every sample data, Five common features were extracted from each sensor response as follows: (1) the maximum absolute response value: $R_{max}=max\left(|R|\right)$; (2) the integral value between the response curve and X axis: $R_{int}=\int_{0}^{T}R\left(t\right)dt$; and (3–5) the maximum value of exponential moving average of derivative of R: $E_{a}\left(R\right)=max\left(|y\left(k\right)|\right)$, k∈[1,400]. The exponential moving average is defined as y(k)=(1−a)y(k−1)+a(R(k))−R(k−1) with smoothing factors a=0.005,0.05,0.5 (using three different *a* to get three different features). Since there are 16 sensors in the E-nose, 80 features were obtained for each sample to make analysis. These features reflect the differences in signal for different samples such as the sensitivity and saturability as performed by sensors.

### 3.3. Aggregated Conformal Prediction

In our early work, we applied conformal prediction (CP) to make E-nose analysis. An optimized framework named inductive conformal prediction (ICP) was also presented to improve efficiency of CP [53,54]. In this study, a reliable machine learning framework named aggregated conformal prediction (ACP) was introduced to deal with the classification problem of E-nose. ACP is an extension of CP and ICP, and it uses several different inductive conformal predictors to make prediction for the same test sample and get the final prediction result by aggregating all their inductive predictions.

For the classification problem in this work, there were two measurable spaces: the *object space* represented as X and the *label space* represented as Y. Every example $z_{i}=\left(x_{i},y_{i}\right)$ can be described by its object $x_{i}\in X$ and its true label $y_{i}\in Y$. Now, we have a set of examples $\left\{z_{1},z_{2},\ldots ,z_{l}\right\}$ with both objects and labels given, and a test example $z_{l+1}$, which we only know the object $x_{l+1}$. To find the true label $y_{l+1}$ of this test example, in the perspective of conformal prediction, we need to try all possible labels c∈Y for the test object to see how well every label conforms to the existing whole set. In the ICP method, the available dataset $\left(z_{1},z_{2},\ldots ,z_{l}\right)$ is divided into two different sets. One set is the training set $\left\{z_{1},\ldots ,z_{m}\right\}$, which is used to train a model by use of the underlying algorithm and calculate *nonconformity score* α for all the other examples. The other set is the calibration set $\left\{z_{m+1},\ldots ,z_{l}\right\}$, which is used to calculated a *p*-value for the possible labels by comparing nonconformity scores of the examples included in. Every possible label c∈Y for the test object is assigned with the *p*-value to evaluate how well the test example combining with the current label conforms to whole set. The *p*-value is defined in the following manner:

(2) $$\begin{array}{c} p^{c}=\frac{|\{j=m+1,\ldots ,l|\alpha_{j}\geq \alpha_{l+1}^{c}\}|+1}{l-m+1} \end{array}$$

where

(3) $$\begin{array}{cc} \alpha_{j} & :=A(\left\{z_{1},\ldots ,z_{m}\right\},z_{j}),j=m+1,\ldots ,l \end{array}$$

(4) $$\begin{array}{cc} \alpha_{l+1}^{c} & :=A(\left\{z_{1},\ldots ,z_{m}\right\},\left(x_{l+1},c\right)) \end{array}$$

*A*, called *nonconformity measure*, depends on the underlying algorithm used to build the classification model. Then, if a *significance level*ϵ∈(0,1) (1−ϵ is known as the confidence level [53]) were chosen, we would get the prediction region that contains all possible labels under the following circumstance:

(5) $$\begin{array}{c} \Gamma^{\epsilon }\left(z_{1},\ldots ,z_{l},x_{l+1}\right):=\left\{c|p^{c}>\epsilon \right\} \end{array}$$

ICP uses a part of available data as training set and calibration set, which may lead to less powerful model and high variance. To overcome these drawbacks, some methods, such as *cross conformal prediction* and *bootstrap conformal prediction*, are proposed, and ACP is a generalization of them [47,48]. In the ACP method, the following procedure is repeated for *K* times: extract a training set of sample $Q_{k}:=\left\{z_{1_{k}}^{*},\ldots ,z_{n_{k}}^{*}\right\}\left(k=1,\ldots ,K\right)$ from the available data $\left\{z_{1},z_{2},\ldots ,z_{l}\right\}$ using a *consistent resampling* procedure defined by Carlsson [47]. For the test object $x_{l+1}$, the p-value is computed every time using ICP method:

(6) $$\begin{array}{c} p_{k}^{c}=\frac{|\{z_{j}\in \left\{z_{1},z_{2},\ldots ,z_{l}\right\}\backslash Q_{k}|\alpha_{j}\geq \alpha_{l+1}^{c}\}|+1}{T+1} \end{array}$$

where T is the size of $\left\{z_{1},z_{2},\ldots ,z_{l}\right\}\backslash Q_{k}$ and

(7) $$\begin{array}{cc} \alpha_{j} & :=A\left(\left\{z_{1_{k}}^{*},\ldots ,z_{n_{k}}^{*}\right\},z_{j}\right),z_{j}\in \left\{z_{1},z_{2},\ldots ,z_{l}\right\}\backslash Q_{k} \end{array}$$

(8) $$\begin{array}{cc} \alpha_{l+1}^{c} & :=A(\left\{z_{1_{k}}^{*},\ldots ,z_{n_{k}}^{*}\right\},\left(x_{l+1},c\right)) \end{array}$$

Finally, we gather all the $p_{k}^{c}\left(k=1,\ldots ,K\right)$ and define the ACP *p*-values as follows:

(9) $$\begin{array}{c} p^{c}=\frac{1}{K}\sum_{k=1}^{K}p_{k}^{c}\;k=1,\ldots ,K \end{array}$$

We can also get the prediction region by setting ϵ and using Equation (5).

The underlying algorithm, which decides the nonconformity measure, has great effects on the informational efficiency (the size of prediction region) of the conformal predictor. In this paper, we define ICP-method and ACP-method as the combination of the framework and underlying method.

In conformal prediction, there are two important indicators to estimate the reliability of every prediction. One is called *confidence* and the other is called *credibility*. The two indicators are defined as follows:

(10) $$\begin{array}{cc} confidence & :sup\{1-\epsilon :|\Gamma^{\epsilon }|\leq 1\} \end{array}$$

(11) $$\begin{array}{cc} credibility & :inf\{\epsilon :|\Gamma^{\epsilon }|=0\} \end{array}$$

For classification tasks, confidence equals 1 min the second highest *p*-value. Confidence shows how much we believe in this prediction, thus it should be as high as possible for an excellent predictor. Credibility equals the highest *p*-value, and a low credibility (close to 0) represents that the test sample is atypical. Thus, credibility should not be too low. The two important indicators can give us some information about the reliability for each prediction, thus they should be reported when evaluating the predictor. We can also analysis the indicators when using ICP and ACP predictors.

## 4. Results and Discussion

### 4.1. Comparison of Different Conformal Predictors and Simple Predictor

**Figure 4:**
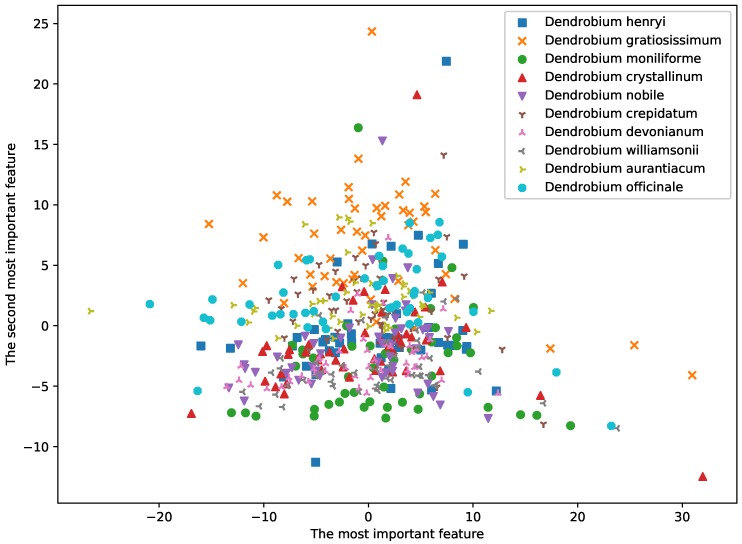
Distribution of samples using PCA method.

In this study, we used ICP and ACP predictors to analyze the E-nose data of all 500 samples from 10 species of dendrobium (50 samples for each specie) in offline mode. At the beginning of analysis, principal component analysis (PCA) was used to depict the distribution of the samples by lowering the dimension to 2D space in Figure 4. The variances of the two PC were 48.42 and 23.27, which accounted for 37.75% and 18.14% of total variance.

To evaluate all the predictors, 10-fold cross-validation was chosen, which is performed as follows: Divide the dataset into 10 parts averagely and every parts contains the same number of samples from each specie of dendrobium. Take nine parts as a training set and the remaining as a testing set to make evaluation. Repeat the cycle 10 times until all parts have been treated as testing set once. For ACP method, bootstrapmethod was used to sample the data. The parameter of K is of great significance to it, which is related to the size of training set and calibration set, and also has influences on the variance and resource consumption for ACP model. After testing, finally, the K was set to be 5 considering the balance among all aspects. Two different machine learning methods were chosen as the underlying algorithms, which have often been used in conformal prediction [47]: (1) support vector machine (SVM) with a radial basis kernel function (the penalty term was set as C = 6000 and the gamma for radial basis kernel function was set as gamma = 0.001); and (2) random forest (RF) with 500 trees.

To assess the classification accuracy, we set a ϵ to force the conformal predictors output one label having the highest *p*-value at each time. This method is called *forced prediction* [45]. Simple machine learning methods without combining conformal prediction framework were also used to make comparison, were defined as simple predictions. The mean value of the 10-fold cross-validation classification accuracy was obtained to make comparisons. Table 3 shows the results of different predictors.

According to the results, it is clear that all the predictors obtained classification accuracy above 70% and ACP obtained nearly 80%. In other articles, MS reaches an accuracy from 83.1% to 88.4% and the accuracy of IRS reaches 88.7% for the classification of six different species [22]. When two-dimensional near-infrared (2D-NIR) is used to classify three different dendrobiums, the error rate is lower than 10% [26]. Although the accuracy was only close to 80% in our work, more species were analyzed here and it was enough to assist pharmacist to make determination with low cost and short time. It proved to be a valuable method to use E-nose and machine learning method to classify these 10 different species of dendrobium. Compared with other predictors, ICP had a classification accuracy approximately 6% lower than simple predictors because ICP only used part of training set to train the model and the remaining data to calculate the *p*-values. Meanwhile, ACP obtained an improvement of about 2% when compared with simple predictors and 8% with ICP because ACP used bootstrap method to sample the data five times for building different models, which reduced the variance of training data. However, ACP spent five times the resources as ICP to build the five models to make predictions, which is an important disadvantage of ACP.

Without too much sacrifice of accuracy, conformal predictors also have obvious advantages in contrast with other predictors. When ICP and ACP predictors were used, the information about reliability for every prediction result could be gained. For example, when predicting the gas sample No. 45, the outputs of different predictors using SVM as underlying algorithms are shown in Table 4 (the output numbers represent the different possible labels *c* which mentioned in Section 3.3).

We can see ICP and ACP gave p-value as assessments of reliability to all the possible labels for the current sample, rather than just give one predicted result making the prediction more comprehensive. The two indicators confidence and credibility were calculated from the *p*-values to make evaluation. From the results of No. 45 sample, we can see both conformal predictors provided high confidence values (close to 1), which means they were of great confidence to make such prediction, and ICP-SVM was more confident than ACP-SVM. Meanwhile, both credibility values were not too low, showing that No. 45 sample was not too atypical in the models. Admittedly, analysis with conformal predictors still has disadvantages. The major disadvantage of conformal predictors is that they call for more resources consumption. Because conformal predictors make predictions by analyzing every possible label and must calculate nonconformity scores for a portion of the training samples, they may take more time and use more resource compared with other predictors under the same conditions. However, this advantages could be ignored when the training set is small and the equipment works well. Thus, it can be demonstrated that ICP may sacrifice some classification accuracy compared with simple prediction, while ACP even has an improvement, and both of the conformal predictors provide reliability information.

### 4.2. Validity and Efficiency of Conformal Prediction

**Figure 5:**
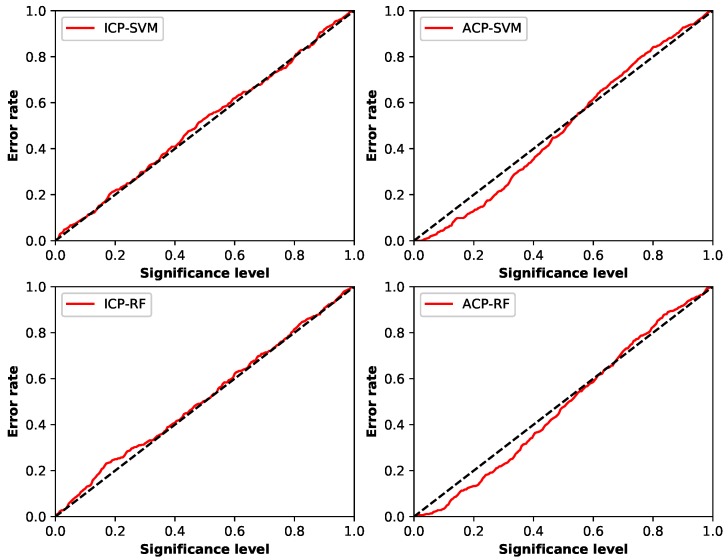
Calibration plot of different conformal predictors.

There are also two main indicators to assess quality of conformal predictors. One is called validity and the other is efficiency [45,53]. Validity focuses on how reliable the predictors are, which depends on whether the error rate is always less or equal to the preset significance level ϵ. Error rate is the ratio of the total number of the test samples that are predicted $\Gamma^{\epsilon }$ not including the true label in chosen ϵ to the number of all test samples [48]. One common method to check the validity of a predictor is using calibration plot mapping each significance level ϵ∈(0,1) to the percentage of erroneous predictions made by the set predictor $\Gamma^{\epsilon }$ on the whole test set [48]. Figure 5 is the calibration plots of the different conformal predictors in this experiment.

From the calibration plots, good calibration in this experiment is shown when using ICP framework. The error rate of ICP-SVM was generally equal to the significance level, and that of ICP-RF was not too often higher than the significance level when ϵ was less than 0.4. ACP framework was also well calibrated, for the error rates of both ACP-SVM and ACP-RF Were only slightly higher than ϵ when it was above 0.6. Comparing these two frameworks, we found ACP was better calibrated when ϵ was under 0.6 and ICP preformed better in the rest of situation, and they both had good validity.

**Figure 6:**
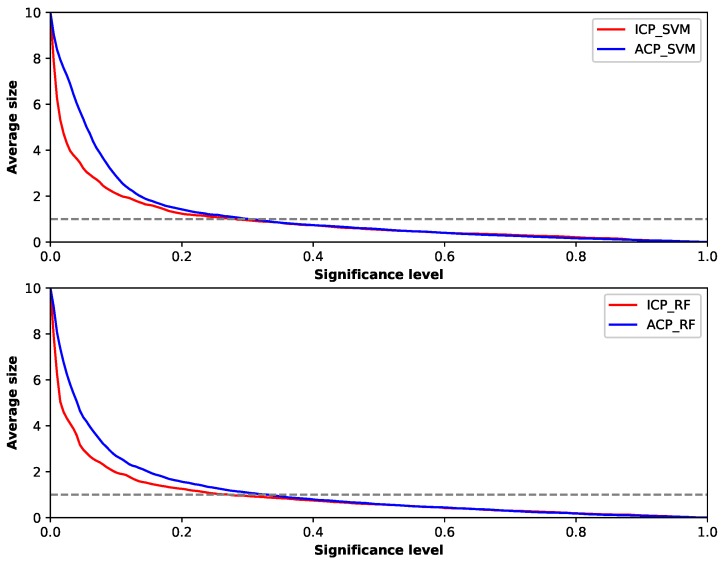
Average size of prediction set for different conformal predictors.

Since the output of conformal prediction is not just a label but a set of labels, the size of the set is important. The efficiency of prediction is related to the size of prediction set and reflects how informative the predictors are. To make the prediction more efficient, the size of prediction set needs to be as small as possible. We can check the efficiency of different predictors by examining the average sizes of their outputs with different significance levels in Figure 6.

The top picture in this figure shows the result of ACP and ICP using SVM as the underlying method, and the bottom picture shows the results of using Random Forest as the underlying method. Although different underlying methods were used, the two conformal predictors performed similarly. We found that, when using ACP framework, the mean output sizes were bigger than those using ICP framework at a low level of ϵ, reflecting ICP had better efficiency than ACP in that situation in this experiment. Additionally, it explains why the error rate of ACP was lower than that of ICP with low level of ϵ shown in the calibration plots, for the output contained more possible labels. However, as ϵ increased, the mean output sizes of ACP and ICP got closer and became under one label almost at the same time. Since it is of no value for conformal prediction if the ϵ is too low, the ACP was not worse than ICP in efficiency aspect. From the discussion above, we can conclude that ACP obtained a higher accuracy, the same validity and a not too much lower efficiency than ICP.

### 4.3. Confidence and Credibility of Conformal Predictors

As mentioned above, confidence and credibility are two important indicators reflecting the reliability of every prediction made by conformal predictor. The overall levels of the two indicators can be used for performance assessment of conformal predictors. Table 5 shows the overall levels of these two indicators using different predictors.

High confidence level is a sign of informational efficiency, and credibility level being not too low is a sign of validity. We can see ICP got a higher mean confidence level than ACP in this experiment, which proved that the efficiency of ICP was better than ACP. All the mean credibility levels being not too low reflected good calibration when using different predictors. The overall levels of the two indicators is another approach to evaluate the predictors. The analysis result from the two indicators conformed well to the results presented in Section 4.2.

## 5. Conclusions

In this work, an approach to discriminate different species of dendrobium used as Chinese medicine with a self-assembled electronic nose was elaborated, and aggregated conformal prediction was applied to analyze E-nose data. E-nose was proven to be a fast and valid method to discriminate dendrobium with a classification accuracy near 80%. We also found that aggregated conformal prediction is of great value to analyze E-nose data as it provides reliable information of every possible label with high accuracy. Aggregated conformal predictor was also compared with inductive conformal predictor and ACP obtained a higher accuracy than ICP with an average improvement of 6.2% when using different underlying algorithms. Validity and efficiency, which are two main indicators to assess quality of conformal predictors, were also discussed and both frameworks were proven to be well calibrated and efficient in this experiment. Although at a sacrifice of efficiency to make more accurate prediction at a low ϵ, ACP did not have too much loss in that situation, which indicates the potential of this framework to be used for E-nose analysis. In the future, research that combines E-nose with advanced spectrometer to discriminate different species of dendrobium is promising. Future work should also be encouraged to focus on optimizing nonconformity measures to improve the classification accuracy and using aggregate conformal prediction in online mode to make the model more robust.

## Figures and Tables

**Table 1:** Details of the dendrobium species.

Name of Specie	Place of Production
*Dendrobium henryi*	Yunnan, China
*Dendrobium gratiosissimum*	Yunnan, China
*Dendrobium moniliforme*	Yunnan, China
*Dendrobium crystallinum*	Yunnan, China
*Dendrobium nobile*	Yunnan, China
*Dendrobium crepidatum*	Yunnan, China
*Dendrobium devonianum*	Zhejiang, China
*Dendrobium williamsonii*	Yunnan, China
*Dendrobium aurantiacum*	Zhejiang, China
*Dendrobium officinale*	Zhejiang, China

**Table 2:** The Target gases of sensors.

No.	Sensor Name	Target Gases	Optimal Detection Concentration
S1	TGS800	Carbon monoxide, ethanol, methane, hydrogen, ammonia	1–30 ppm
S2	TGS813	Carbon monoxide, ethanol, methane, hydrogen, isobutane	500–10,000 ppm
S3	TGS813	Carbon monoxide, ethanol, methane, hydrogen, isobutane	500–10,000 ppm
S4	TGS816	Carbon monoxide, ethanol, methane, hydrogen, isobutane	500–10,000 ppm
S5	TGS821	Carbon monoxide, ethanol, methane, hydrogen	30–1000 ppm
S6	TGS822	Carbon monoxide, ethanol, methane, acetone, n-Hexane,	50–5000 ppm
		benzene, isobutane	
S7	TGS822	Carbon monoxide, ethanol, methane, acetone,	50–5000 ppm
		n-Hexane, benzene, isobutane	
S8	TGS826	Ammonia, trimethyl amine	30–300 ppm
S9	TGS830	Ethanol, R-12, R-11, R-22, R-113	100–3000 ppm
S10	TGS832	R-134a, R-12 and R-22, ethanol	100–3000 ppm
S11	TGS800	Carbon monoxide, ethanol, methane, hydrogen, ammonia	1–30 ppm
S12	TGS2620	Methane, Carbon monoxide, isobutane, hydrogen	50–5000 ppm
S13	TGS2600	Carbon monoxide, hydrogen	1–30 ppm
S14	TGS2602	Hydrogen, ammonia ethanol, hydrogen sulfide, toluene	1–30 ppm
S15	TGS2610	Ethanol, hydrogen, methane, isobutane/propane	500–10,000 ppm
S16	TGS2611	Ethanol, hydrogen, isobutane, methane	500–10,000 ppm

**Table 3:** Classification accuracy of different predictors.

Underlying Method	Framework		
	Simple	ICP	ACP
SVM	77.4%	70.85%	79.4%
RF	76.65%	71.75%	78.00%

**Table 4:** Output of different predictors for No. 45 sample.

Predictors	Output						Confidence	Credibility
	1	2	3	4	...	10		
**SVM**	False	False	False	False	...	True	-	-
**ICP-SVM**	0.007	0.009	0.012	0.007	...	0.813	0.915	0.813
**ACP-SVM**	0.009	0.007	0.040	0.012	...	0.765	0.854	0.765

**Table 5:** The overall levels of confidence and credibility using different conformal predictors.

Conformal Predictor	Confidence		Credibility	
	Mean	ST.dev.	Mean	ST.dev.
ICP-SVM	0.908	0.092	0.515	0.252
ACP-SVM	0.885	0.094	0.506	0.241
ICP-RF	0.904	0.090	0.535	0.244
ACP-RF	0.877	0.099	0.548	0.232
